# Investigation on the Deformation and Surface Quality of a Bearing Outer Ring during Grinding Processing

**DOI:** 10.3390/mi15050614

**Published:** 2024-04-30

**Authors:** Jiang Guo, Pengyu Long, Yong Zhao, Haojie Xu, Zhaoyuan Yang, Jianjun Wang, Tingting Li, Jiwu Tang

**Affiliations:** 1 State Key Laboratory of High-Performance Precision Manufacturing, Dalian University of Technology, Dalian 116024, China; 2 Dalian Guangyang Bearing Company Limited, Dalian 116602, China; 3 College of Applied Technology, Dalian Ocean University, Dalian 116086, China

**Keywords:** thin-walled bearing, grinding, machining quality, machining deformation, deformation mechanism

## Abstract

Thin-walled bearings are widely used owing to the advantages of their light structure, high hardness, and strong load-carrying capacity. However, thin-walled bearings are often prone to deformation during the machining process, which can seriously affect the performance of the bearings. In addition, the machining deformation and quality of bearings are difficult to balance. To address the above issues, this paper investigates the effects of the machining parameters on the machining deformation, surface quality, and machining efficiency of a thin-walled bearing during the roughing stage. The dynamic balance between deformation inhibition and high quality in rough grinding was studied, and the optimal parameters for thin-walled bearing outer ring grinding were obtained. The deformation mechanism of thin-walled bearings caused by grinding was revealed through simulation and experimental analysis. The results show that the machining deformation and quality reach a balance when the workpiece speed is 55 r/min, the grinding wheel rotational speed is 2000 r/min, and the feed rate is 0.1 mm/min. Deformation increases with the increase in workpiece speed and grinding wheel speed. At the same time, the surface roughness increases with the increase in the workpiece speed, but the increase in the wheel speed will improve the surface roughness. As the workpiece speed increases, the surface topography shows a more pronounced stockpile of material, which is ameliorated by an increase in grinding wheel speed. As the rotational speed of the workpiece increases, the number of abrasive grains involved in the process per unit of time decreases, and the surface removal of the workpiece is less effective, while the increase in the rotational speed of the grinding wheel has the opposite effect. The grinding deformation of thin-walled bearings is mainly induced by machining heat and stress. As the rotational speed increases, the heat flux in the grinding zone increases. More heat flux flows into the surface of the workpiece, causing an increase in thermal stresses on the inner surface of the bearing collar, leading to greater deformation. The temperature in the grinding area can be reduced during machining, realizing a reduction in deformation. The research content contributes to the balance between high quality and low distortion in machining processes.

## 1. Introduction

The grooves of the outer ring of the bearing are in frequent contact with the rolling body and are subject to vibration and collision, etc. The surface quality and processing accuracy have a greater impact on the rotary accuracy and service life of the outer ring of the bearing. As a starting point for the topic, bearing processing efficiency and quality directly affect the performance of the host and the economic benefits of the manufacturer [1]; an efficient machining process and good machining quality have a decisive impact on the overall performance of the bearing. High-precision bearings are the core components of major equipment and precision equipment, and their reliability has a crucial impact on the service life of mechanical equipment. According to relevant research and analysis, about 30% of mechanical failures are caused by the failure of bearings in rotating parts [2]. With the rapid development of science and technology, bearings are required to overcome difficult conditions such as high speed, heavy load, strong impact force, and high-frequency friction, and they cannot be prone to wear, cracking, corrosion, and other defects, to avoid damage to machinery and equipment and safety accidents [3].

Grinding and lapping are commonly used in machining. Fan et al. analyzed the effect of sodium silicate as well as SHMP on the grinding effect of muscovite and characterized its surface roughness, etc. [4,5]. Nowadays, the outer ring of bearings is mainly processed by grinding. Li Xin et al. proved that the radial feed rate among the grinding parameters has the greatest influence on the radial wear of the grinding wheel, and the grinding wheel rotational speed has the greatest influence on the surface roughness of the workpiece [6]. Tang et al. analyzed the structural characteristics and processing difficulties of the outer ring of cylindrical roller bearings with a three-valve wave raceway and adjusted the grinding process [7]. YAMADA et al. compared the surface quality of workpieces after grinding with different grinding wheels to study the effect of grinding wheels with different contact stiffness on the surface quality and found that the surface roughness decreases with the decrease in the contact stiffness of the grinding wheel [8]. The research on deformation defects in the process of bearing processing involves multi-disciplinary theories, including material mechanics [9], theoretical mechanics [10], and machining technology [11], etc. It is necessary to conduct targeted research on different processes and different types of bearing parts. NIK et al. studied the surface roughness of a Ti6Al4V alloy after ultrasonic grinding and normal grinding, and the results showed that ultrasonic grinding can effectively reduce the surface roughness and improve the surface quality [12]. Tong et al. proposed a new calculation program for the grinding process of bearings, which incorporated the deformation of the rolling body into the design model, and analyzed the internal load distribution and the displacement of the inner ring based on it. The research results show that the deformation of the rolling element has an important influence on the load of the bearing, and an increase in the load will cause a large deviation in the axial displacement of the bearing, which will make the bearing prone to failure and other consequences [13].

The grinding process of the outer ring of the bearing collar is carried out on a centerless grinding machine, and in the centerless grinding process, if the grinding parameters are not properly selected, it can lead to serious deformation [14]. The main technical indexes of bearing outer ring grinding processing include surface roughness, ellipticity, inner diameter size, wall thickness difference, vibration pattern and burns, etc., and the deformation and surface roughness are also important factors affecting the performance of bearings [15,16]. Processing quality is mainly concerned with two aspects: on the one hand, the processing-induced deformation of the outer ring, which is mainly reflected by the two parameters of dimensional change and ellipticity change; on the other hand, the surface quality, which is analyzed by the roughness change brought about by grinding. The relationship between outer ring deformation and residual stress is also analyzed through simulation. The research content of the article helps to achieve a balance between high surface quality and reduced deformation. The study realizes the reduction in deformation during the machining process which is instructive for practical machining and can improve the production efficiency of enterprises.

## 2. Experimental Design and Simulation Modeling

### 2.1. Experimental Setup

Through the preliminary research, this study carries out experiments for the grinding processing of the outer ring of NCF1856V.01 model bearings. The material used was GCr15 steel, which is an economical and practical high-carbon chromium bearing steel, with its high hardness, high toughness, high bearing capacity, and other excellent characteristics maintaining the steel market share of this material at a high level. Due to its high carbon content, GCr15 steel is easier to cut, process, and carry out hot work with, and it can be processed into various shapes of precision parts, which are widely used in the manufacturing of various bearing rings and rolling parts. The material properties are shown in Table 1.

**Figure 1:**
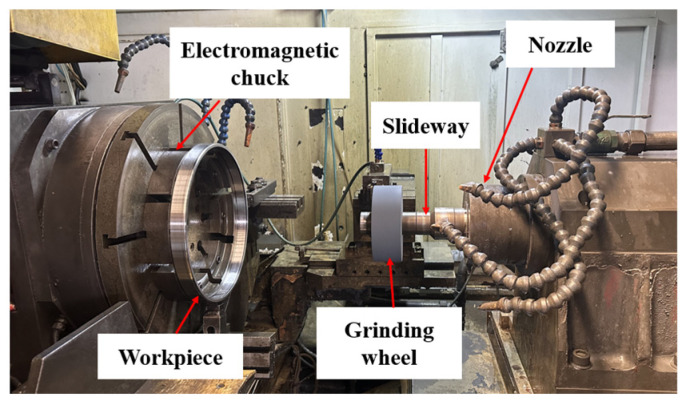
Schematic diagram of processing area.

The machine tool used for this grinding machining experiment is a CNC-tapered and cylindrical roller bearing outer ring raceway and inner ring bore grinding machine of model 3MB2050CNC (Puyang Beain CNC Machinery and Equipment Co., Ltd., Puyang, China), as shown in Figure 1. The machine tool can be used for grinding and finishing the raceways of the outer ring and the inner diameter of the inner ring of tapered and cylindrical roller bearings, and it belongs to the special processing machine tools for bearings.

In this experiment, the machined surface topography and surface roughness were examined with a ZYGO (NewView™ 9000, Zygo Corporation, Middlefield, CT, USA) 3D optical surface profiler and a MarSurf XC 20 mit PCV 200 profilometer (Mahr, Broadway Cresskill, NJ, USA).

### 2.2. Experimental Design

In the process of grinding, a design is mainly affected by three processing parameters: workpiece speed, grinding wheel speed, and feed rate. The roughness of the workpiece surface is measured before and after grinding. When observing the surface topography, besides observing the image, the variation trend of the 3D topography height difference is also reflected according to *S*_z_ (maximum height) in the measured results.

In order to study the influence of workpiece speed, grinding wheel speed, and feed rate on the deformation and surface quality of the outer ring of the bearing, a total of nine groups of machining experiments were designed. The experimental parameters of each group are shown in Table 2.

### 2.3. Numerical Simulation Modeling

A simulation analysis is carried out for the dimensional deformation caused by grinding processing on the bearing outer ring. In this chapter, the ABAQUS 2021 software finite element simulation software is used to calculate the thermal stress in the heat transfer analysis, and then the static general analysis step is adopted. The three-dimensional physical model of the NCF1856V.01 thin-walled bearing outer ring used in the experiment is established in the simulation, and parameters such as heat flux and contact length during grinding are calculated. After the calculation is completed, the grinding temperature finite element calculation is carried out, including the definition of elements and material properties, mesh division, time step determination, loading and solving of moving triangle heat source, and so on. The influence trend of temperature on deformation and the influence of machining parameters on residual stress were analyzed.

The common moving heat source loading models set for numerical simulation of grinding temperature field in existing research mainly include rectangles, trapezoid, triangles, right triangles, parabolas, circular arcs, variable sections, unequal triangles, and so on. In this paper, the triangular moving heat source is adopted, and Dflux is used to design the size and moving mode of the heat source.

**Figure 2:**
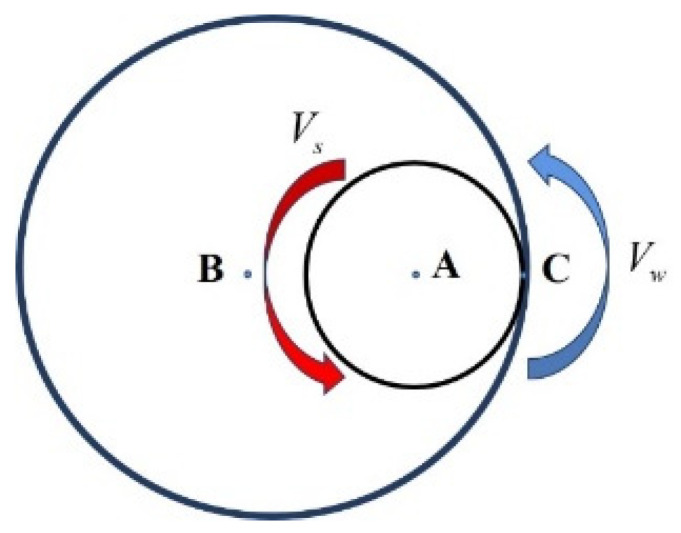
Movement diagram of grinding wheel and bearing outer ring.

In order to simulate the movement of the contact area in the process of grinding the inner raceway of the bearing outer ring, it is necessary to calculate its relative motion speed as the loading speed of the moving heat source. The calculation diagram is shown in Figure 2, where point A is the center of the grinding wheel, point B is the center of the outer ring of the bearing to be machined, and C is the theoretical contact point. The grinding mode is smooth grinding, that is, the rotation direction of the grinding wheel is the same as the rotation direction of the ring. The specific legend is as follows:

The relative speed *V_r_* at contact point C is calculated as the formula:

(1) $$\vec{V_{r}}=\vec{V_{c}}-\vec{V_{e}}$$

where *V_c_* and *V_e_* are, respectively, the linear velocity and implicated velocity of point C, and their calculation formulas are as shown below:

(2) $$V_{c}=\frac{1}{2}d_{s}\omega_{s}$$

(3) $$V_{w}=\frac{1}{2}d_{w}\omega_{w}$$

The effect of residual stresses on deformation is explored by full thermal coupling through ABAQUS 2021 software, and the contact length is calculated.

(4) $$V_{w}=2\pi \cdot R_{w}\cdot n_{w}$$

(5) $$V_{s}=2\pi \cdot R_{s}\cdot n_{s}$$

(6) $$L_{k}=(1-\frac{V_{w}}{60V_{s}})\cdot {\left(\frac{d_{s}\cdot d_{w}\cdot a_{p}}{d_{w}-d_{s}}\right)}^{\frac{1}{2}}$$

where *V_w_* is the workpiece line speed, *V_s_* is the grinding wheel line speed, and *L_k_* is the contact length. *d_s_* is the wheel diameter, *d_w_* is the workpiece diameter, and *a_p_* is the depth of cutting.

In the study of Jin Guangdi et al., the workpiece heat transfer density is obtained as follows [18]:

(7) $$q_{w}=\frac{\varepsilon_{\mathrm{ws}}\left(q-q_{\mathrm{ch}}\right)}{1+\varepsilon_{\mathrm{ws}}h_{f}/h_{w}}$$

Accordingly, *q_ch_* is the heat transfer of chips, and there is powder in the actual processing of chips, hence there are fewer chips, so do not consider the heat composition, that is, *q_ch_* = 0. *h_f_* is the convective heat transfer coefficient of grinding fluid and *h_w_* is the heat transfer coefficient of the workpiece.

In the study, the heat distribution ratio between the grinding wheel and the workpiece is as follows [19]:

(8) $$\varepsilon_{\mathrm{ws}}=\frac{1}{1+\frac{0.97k_{g}}{\sqrt{r_{0}v_{s}{\left(k\rho c\right)}_{w}}}}$$

Accordingly, *k_g_* is the heat conduction coefficient of grinding wheel grains, *k_w_* is the heat conduction coefficient obtained by bearing rings, *ρ_w_* is the density of bearing rings, *c_w_* is the specific heat capacity of bearing rings, and *r*_0_ is the contact radius of grinding wheel grains. In the study of Rowe W et al., *r*_0_ is obtained by being calculated as *r*_0_ = 15 μm [20].

**Figure 3:**
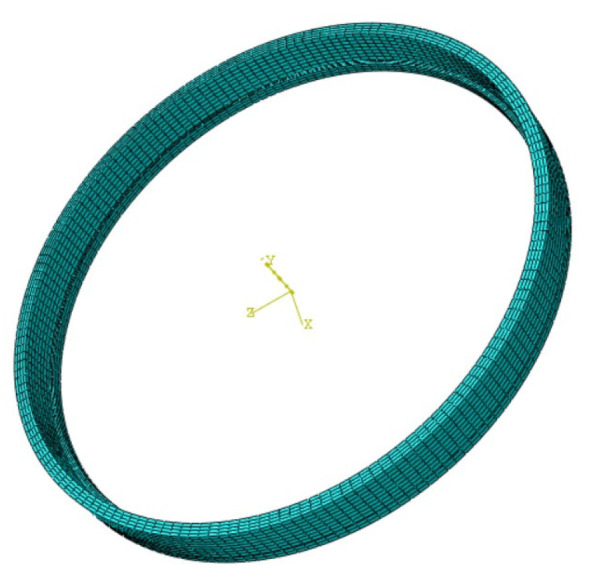
The meshing of bearing outer rings.

Meshing is the division of a geometric surface or cube into a number of small units, according to which the desired variables are calculated using partial differential equations. The size and quality of meshing elements have an important influence on the efficiency of calculation and the reliability of analysis results. If the number of grids divided by the model is small and the coefficients of the grids divided are loose, the calculation results will be inaccurate. If the number of grids is too large, the calculation time of the model may be longer, resulting in software crashes and other results. In this study, a linear hexahedral grid with DC3D8 cell type was set, with a total of 9328 cells and 12,672 nodes, as shown in Figure 3.

In this study, the heat transfer mode of the grinding process was set to be thermal convection, and the initial temperature was set using the heat source subroutine generator (DFLUX) based on ABAQUS 2021 software to set the size of the triangular heat source and the way of moving, etc.

## 3. Results and Discussion

### 3.1. Relationship between Grinding Processing Parameters and Deformation

After grinding, the change in the inner diameter size and the change in the outer ring ellipticity were measured to reflect their influence on the regularity of grinding parameters on deformation. The processing quality will directly affect the service life and performance of the bearing [21,22]. To reduce experimental error, three outer rings are processed for each group of machining parameters. Before and after machining, measure the inner diameter size and ellipticity of each group of three outer rings, and take its average value.

**Figure 4:**
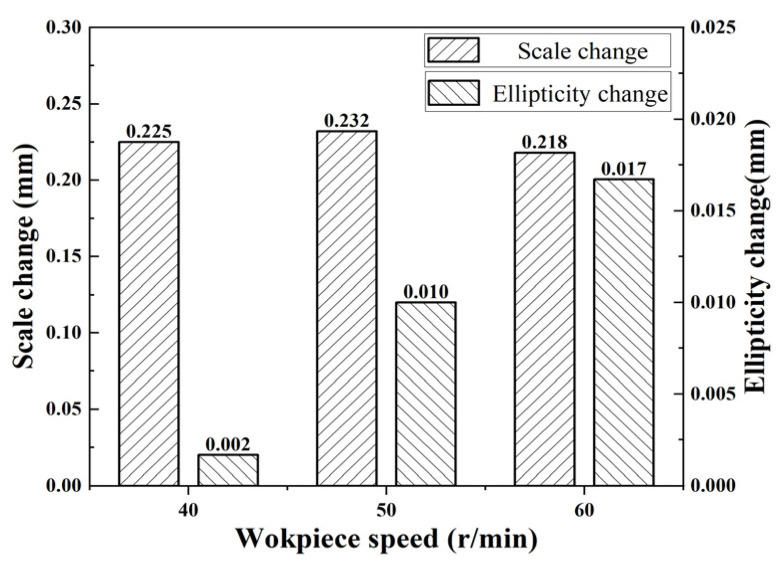
Relationship between workpiece speed and deformation.

Through data organization and calculation, the influence of workpiece speed on the change in inner diameter size and ellipticity is shown in Figure 4. Through data organization, it can be found that the dimensional change as well as the change in ellipticity increase with the increase in workpiece speed.

**Figure 5:**
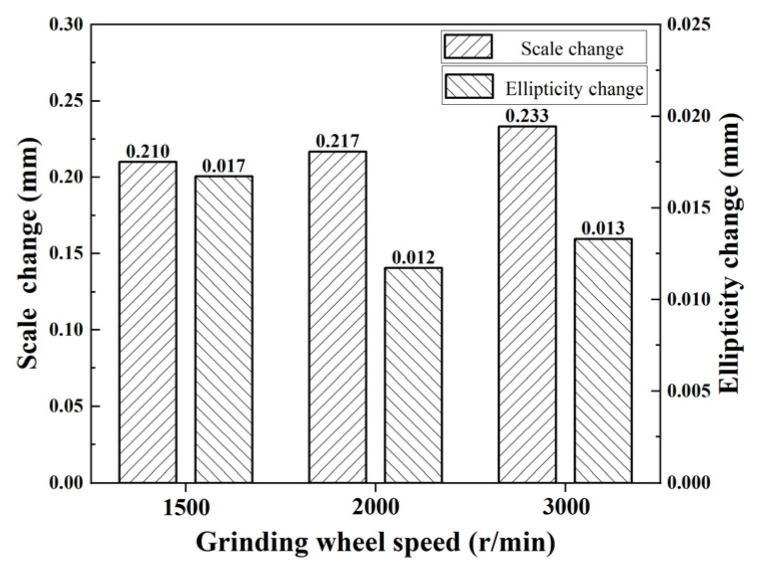
Relationship between grinding wheel speed and deformation.

It can be seen in Figure 5 that the change in inner diameter size increases with the increase in grinding wheel speed, but the change in ellipticity is less affected by the rotational speed of the grinding wheel.

**Figure 6:**
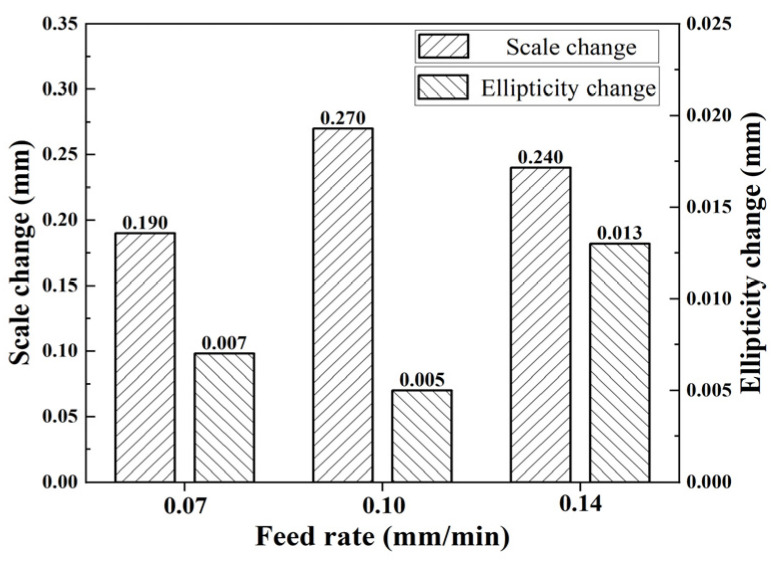
Relationship between feed rate and deformation.

In addition, through data organization and calculation, the effect of feed amount on the inner diameter size and ellipticity change is shown in Figure 6. By analyzing the data, it is known that the change in size and the change in ellipticity are less affected by the feed rate.

### 3.2. Simulation Analysis

**Figure 7:**
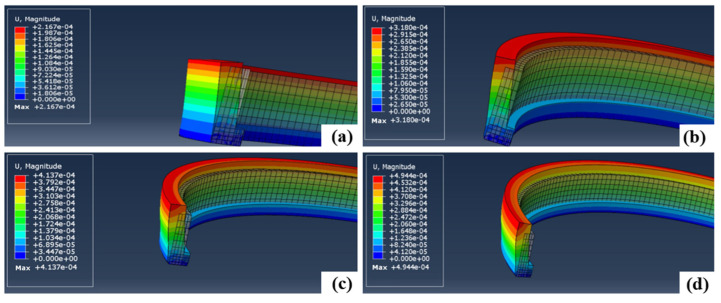
(**a**–**d**) Deformation of the outer ring at different temperatures.

The initial temperatures of the model are 500 °C, 1000 °C, 1500 °C, and 2000 °C, respectively. It can be seen from Figure 7 that the deformation of the outer ring increases with the increase in temperature.

**Figure 8:**
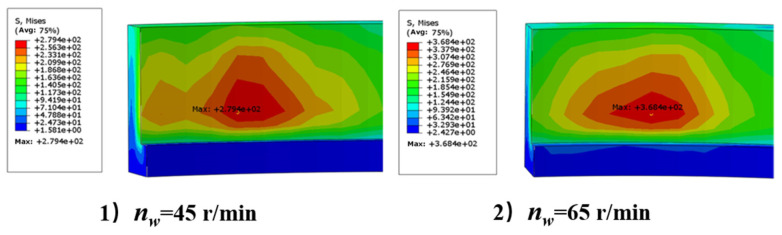
Residual stress under different conditions: (**1**) workpiece speed 45 r/min; (**2**) workpiece speed 65 r/min.

As can be seen from Figure 8, when the workpiece speed increases, the residual stress on the machined surface also increases. The reason for this is that the heat flux in the grinding area increases as the workpiece speed increases. In addition, a high speed usually means that the grinding speed increases, which results in more material being removed. Since grinding is the cutting and friction of the workpiece surface through abrasive particles, higher grinding speeds lead to more heat generation.

**Figure 9:**
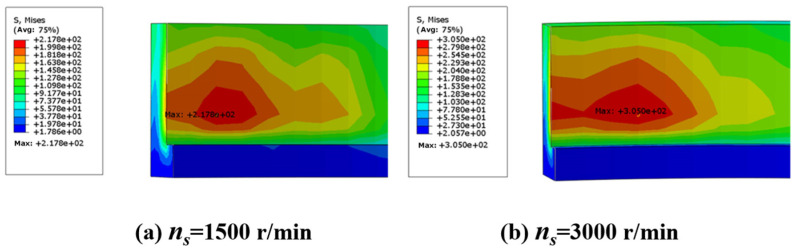
Residual stress under different conditions: (**a**) grinding wheel speed 1500 r/min; (**b**) grinding wheel speed 3000 r/min.

Meanwhile, Figure 9 reflects the regularity of the influence of the grinding wheel speed on the residual stress, and the residual stress on the machined surface also increases with the increase in the grinding wheel speed. The reason is that the high-speed rotating grinding wheel will produce higher friction heat, so the workpiece surface local temperature will increase. When the grinding speed increases, the thermal effect of the grinding area is also enhanced, resulting in a large residual stress.

### 3.3. Analysis of the Deformation Mechanism

**Figure 10:**
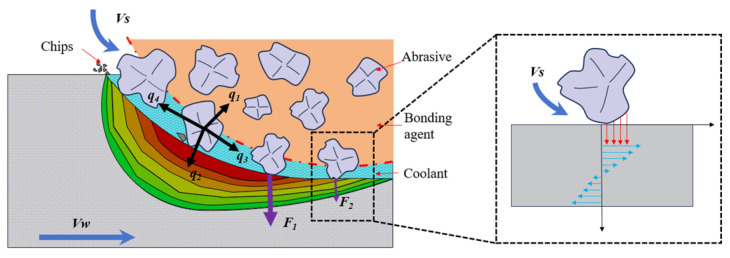
Grinding deformation mechanism.

Experimental and simulation results show that the deformation of the outer ring increases with the increase in both the workpiece speed and grinding wheel speed. As can be seen from Figure 10, the blue arrows represent the speed of motion, the black arrows represent the heat flux distribution, and the purple color represents the stresses. The heat flux generated in the grinding process of the grinding wheel is divided into the heat flux *q*_1_ transmitted to the grinding wheel, the heat flux *q*_2_ transmitted to the workpiece, the heat flux *q*_3_ carried by the coolant, and the heat flux *q*_4_ carried away by the grinding chips, where *q*_2_ is the heat flux *q_w_* calculated by Equation (7). Under the influence of the grinding temperature, the surface layer of the material is subjected to residual tensile stresses, which gradually transform into residual compressive stresses as the depth increases. When the heat flux is large, the thermal stress generated after grinding is also large, resulting in greater deformation of the workpiece.

### 3.4. Relationship between Grinding Processing Parameters and Surface Quality

Roughness is an important index to evaluate the surface quality and micro-geometric errors of machined parts, and different values of surface roughness will have different effects on the fatigue properties of parts. As an important part of the bearing work, the greater the roughness, the smaller the contact area between the bearing channel and the rolling element, and the larger the unit area, the greater the local plastic deformation of the peak, which then affect the working accuracy and vibration resistance of the bearing. Reducing the surface roughness can greatly improve the service life of the bearing.

**Figure 11:**
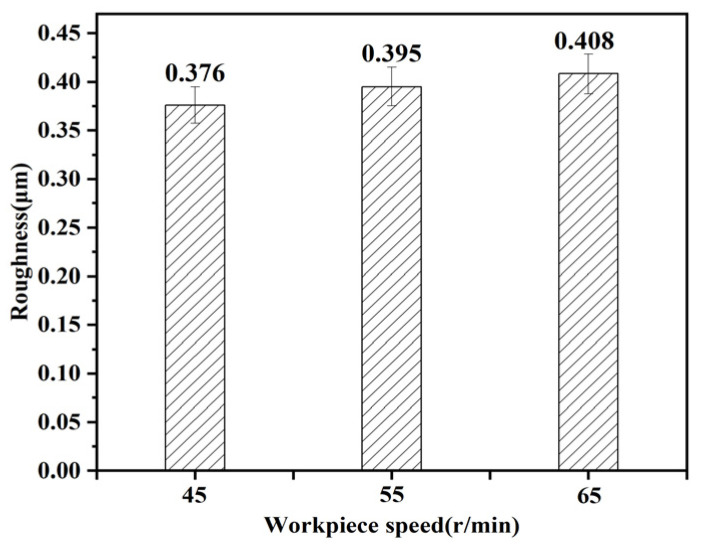
Effect of workpiece speed on roughness.

It can be seen from Figure 11 that the roughness increases with the increase in the workpiece speed. The reason for this phenomenon is that with the gradual increase in the workpiece speed, the number of abrasive particles in the unit time of the grinding wheel decreases, thus increasing the surface roughness value of the machined surface.

**Figure 12:**
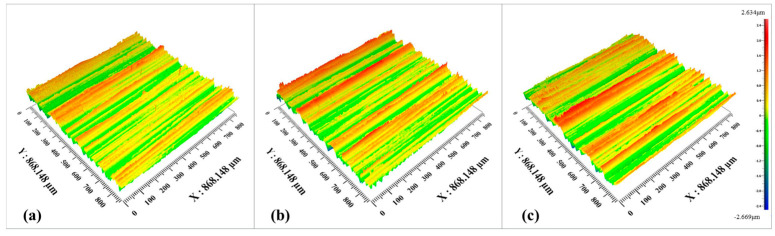
3D morphologies under different workpiece speeds: (**a**) workpiece speed 45 r/min; (**b**) workpiece speed 55 r/min; (**c**) workpiece speed 65 r/min.

**Figure 13:**
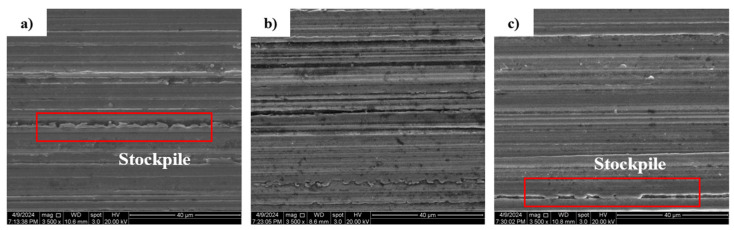
SEM 2D morphology at different workpiece speeds: (**a**) workpiece speed 45 r/min; (**b**) workpiece speed 55 r/min; (**c**) workpiece speed 65 r/min.

In addition, the surface morphology of the machined outer ring gradually deteriorates with the increase in the workpiece speed. In addition, there is an obvious material accumulation phenomenon, and the height difference of the surface topography in the adjacent regions becomes larger. As can be seen in Figure 12 and Figure 13, material removal is carried out on the surface of the workpiece due to the movement of the abrasive grains, and the material stockpile is formed on the surface of the workpiece without abrasive grain movement. The main reason for this phenomenon is the increase in workpiece speed, the reduction in abrasive particles involved in processing per unit time, and the reduction in the material removal rate on the surface of the raceway per unit time, resulting in more material accumulation.

**Figure 14:**
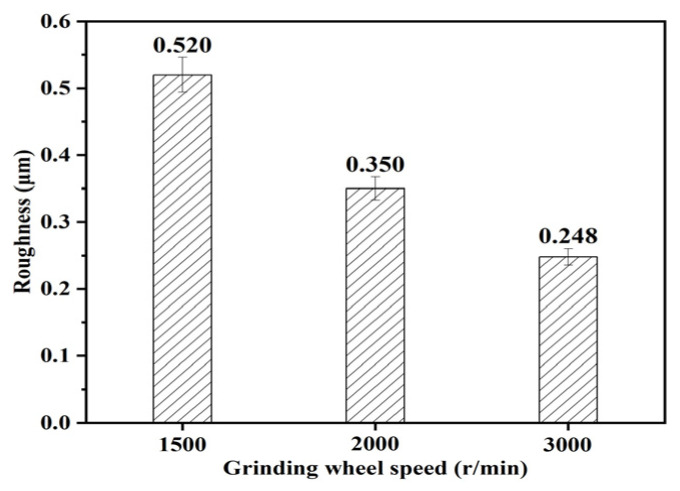
Effect of grinding wheel speed on roughness.

**Figure 15:**
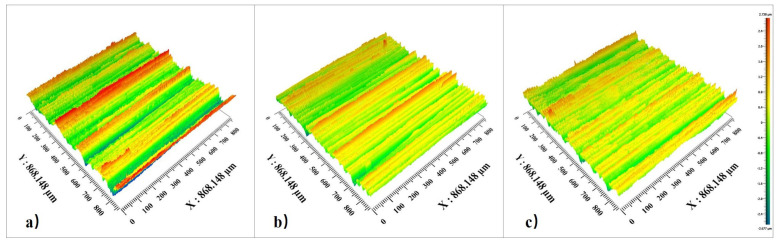
Three-dimensional morphologies under different grinding wheel speeds: (**a**) grinding wheel speed 1500 r/min; (**b**) grinding wheel speed 2000 r/min; (**c**) grinding wheel speed 3000 r/min.

**Figure 16:**
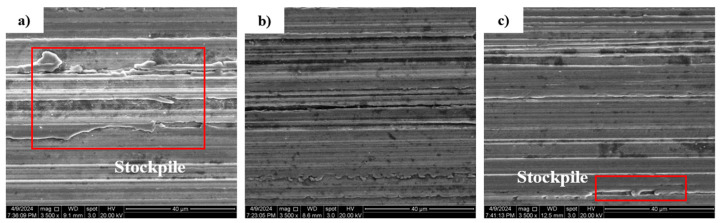
SEM 2D morphology at different wheel speeds: (**a**) wheel speed 1500 r/min; (**b**) grinding wheel speed 2000 r/min; (**c**) grinding wheel speed 3000 r/min.

Similarly, Figure 14 and Figure 15 reflect the pattern of influence of grinding wheel speed on surface roughness and surface topography. It can be seen from Figure 15 that when the workpiece speed and feed rate remain unchanged, the mean value of the inner surface roughness of the outer ring of the thin-walled bearing decreases with the increase in the workpiece speed. The higher the grinding wheel speed, the more abrasive particles pass through the grinding surface per unit of time and the lower the surface roughness. As can be seen in Figure 16, due to the increased number of abrasive grains, the motion trajectory is repeated more, and more material is removed.

**Figure 17:**
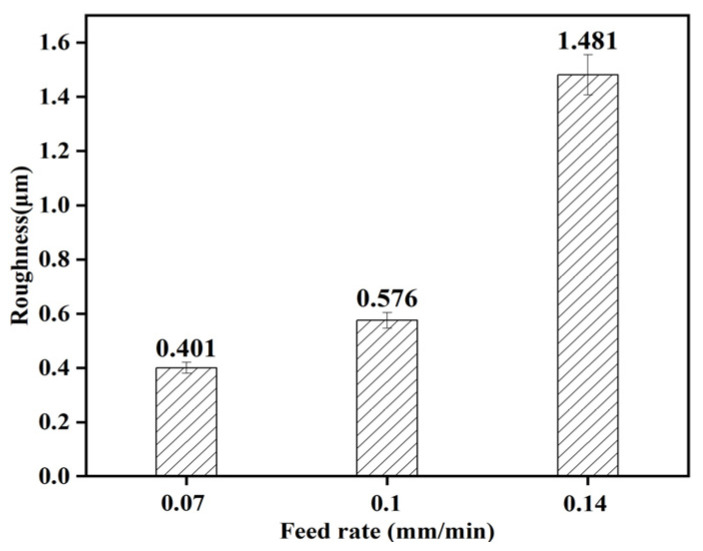
Effect of feed rate on roughness.

**Figure 18:**
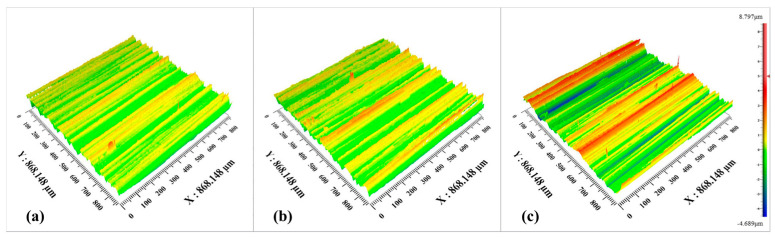
Three-dimensional morphologies under different grinding feed rates: (**a**) feed rate 0.07 mm/min; (**b**) feed rate 0.10 mm/min; (**c**) feed rate 0.14 mm/min.

**Figure 19:**
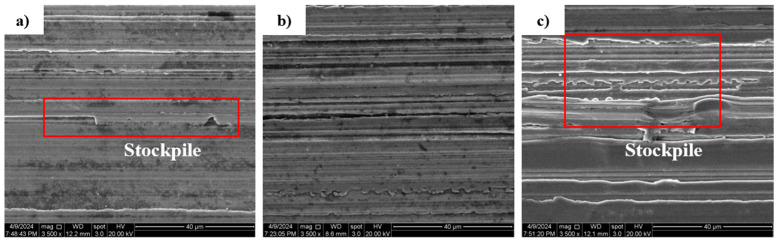
SEM two-dimensional morphology at different feed rates: (**a**) feed rate 0.07 mm/min; (**b**) feed rate 0.10 mm/min; (**c**) feed rate 0.14 mm/min.

From Figure 17, it can be seen that the roughness increases gradually at feed rates of 0.07, 0.10, and 0.14 mm/min, corresponding to 0.401, 0.576, and 1.481 μm, respectively. The roughness increases with the increase in feed rate. The larger the feed, the greater the depth of the abrasive per unit in the wheel plowing the bearing surface and the greater the surface roughness [23]. At the same time, the morphology of the machined surface under different feeding parameters was observed. It can be seen from Figure 18 and Figure 19 that with the increase in the feed rate, the removal of a single abrasive grain increases, the processing contact time between the abrasive grain and the unit area is shorter, the material that is not removed increases, the material buildup phenomenon is more obvious, and the height difference is more obvious, in which the value of *S*_z_ increases from 6.046 μm to 13.485 μm.

## 4. Conclusions

In this paper, the NCF1856V.01 model was used, and grinding processing experiments were carried out using the bearing outer ring, alongside research and analysis of the workpiece speed, grinding wheel speed, and feed volume and how these three influence the regularity of bearing outer ring groove deformation and surface quality. At the same time, using ABAQUS 2021 software on the outer ring of the bearing force, a thermal simulation was undertaken to analyze the effect of residual stress on deformation. The conclusions of the study are as follows:

(1) Through the analysis of the changing trend of the inner diameter and ellipticity before and after the machining experiment, it can be seen that the feed has the least influence on the deformation, and there is no obvious trend in the change in the inner diameter and ellipticity. An increase in workpiece speed and grinding wheel speed will increase the deformation.

(2) With the increase in workpiece speed and grinding wheel speed, the heat flux in the grinding area increases, which leads to the increase in grinding temperature and residual stress on the inner surface of the bearing outer ring. In the study, an increase in workpiece speed resulted in a 31.8% increase in stress, and an increase in grinding wheel speed resulted in a 40% increase in stress. The excessive stress makes the deformation of the outer ring more obvious.

(3) The results show that the average surface roughness of the machined surface increases with the increase in workpiece speed and the feed rate, and the material accumulation will be more serious. In particular, the roughness increased by a factor of 2.7 with increasing feeds. With the increase in grinding wheel speed, the surface roughness becomes smaller.

## Figures and Tables

**Table 1:** Mechanical properties of GCr15 steel [17].

Material Properties	Unit	Numeric
Coefficient of thermal expansion		1.5 × 10^−5^~8 × 10^−5^
Poisson’s ratio		0.3
Modulus of elasticity	MPa	2.06 × 10 × 10^5^
Tensile strength	MPa	≥861.3

**Table 2:** Experimental processing parameters.

Processing Parameters	Groups	Workpiece Speed	Grinding Wheel Speed	Feed Rate
Workpiece speed (r/min)	1	45	2000	0.07
	2	55	2000	0.07
	3	65	2000	0.07
Grinding wheel speed (r/min)	1	55	1500	0.07
	2	55	2000	0.07
	3	55	3000	0.07
Feed rate (mm/min)	1	55	2000	0.07
	2	55	2000	0.10
	3	55	2000	0.14

## Data Availability

Data are available within the article.
